# Serotonin receptors in depression: from A to B

**DOI:** 10.12688/f1000research.9736.1

**Published:** 2017-02-09

**Authors:** Katherine M. Nautiyal, René Hen

**Affiliations:** 1 Division of Integrative Neuroscience, New York State Psychiatric Institute, and Department of Psychiatry, Columbia University, NY, USA; 2 Departments of Neuroscience and Pharmacology, Columbia University, NY, USA

**Keywords:** serotonin, MDD, major depressive disorder, serotonin receptor, 5-HT1A, 5-HT1B, 5-HTT, selective serotonin reuptake inhibitors, antidepressant

## Abstract

The role of serotonin in major depressive disorder (MDD) is the focus of accumulating clinical and preclinical research. The results of these studies reflect the complexity of serotonin signaling through many receptors, in a large number of brain regions, and throughout the lifespan. The role of the serotonin transporter in MDD has been highlighted in gene by environment association studies as well as its role as a critical player in the mechanism of the most effective antidepressant treatments – selective serotonin reuptake inhibitors. While the majority of the 15 known receptors for serotonin have been implicated in depression or depressive-like behavior, the serotonin 1A (5-HT _1A_) and 1B (5-HT _1B_) receptors are among the most studied. Human brain imaging and genetic studies point to the involvement of 5-HT _1A_ and 5-HT _1B_ receptors in MDD and the response to antidepressant treatment. In rodents, the availability of tissue-specific and inducible knockout mouse lines has made possible the identification of the involvement of 5-HT _1A_ and 5-HT _1B_ receptors throughout development and in a cell-type specific manner. This, and other preclinical pharmacology work, shows that autoreceptor and heteroreceptor populations of these receptors have divergent roles in modulating depression-related behavior as well as responses to antidepressants and also have different functions during early postnatal development compared to during adulthood.

## Introduction

The serotonin hypothesis of depression has dominated the field of depression for over four decades ^1^. This theory is centered on the idea that reduced serotonin signaling is a risk factor in the etiology and/or pathophysiology of major depressive disorder (MDD) ^2^. However, the most robust body of evidence for the role of serotonin in depression is the efficacy of increasing extracellular serotonin for the treatment of depression. The discovery that the efficacy of tricyclic antidepressants (TCAs) and monoamine oxidase inhibitor (MAOI) antidepressants was largely due to their serotonergic actions, which prompted the use of serotonin selective reuptake inhibitors (SSRIs), the first among them fluoxetine, to treat depression ^3– 6^. These drugs act at the serotonin transporter (5-HTT, also known as SERT) and cause increases in extracellular serotonin, which is the purported mechanism of action ^6– 8^. Many subsequent drugs inhibiting serotonin reuptake have shown behavioral efficacy as antidepressant drugs, suggesting that increasing synaptic serotonin levels may lead to the treatment of depression ^6, 9^.

Despite the relative success in treating depression by increasing extracellular serotonin, there is a lack of strong evidence supporting a direct correlation between low serotonin signaling and depression. While some studies report an association between levels of platelet serotonin and depression, this has not been a consistent finding in large sample sets, and it is also unclear how platelet levels are related to brain levels of serotonin ^10, 11^. Additionally, few studies report direct correlations between cerebrospinal fluid 5-hydroxyindoleacetic acid (5-HIAA), a serotonin metabolite, and depression ^12, 13^. Low levels of tryptophan have been consistently linked to depression; however, these effects could be independent of serotonin ^14, 15^. The lack of consistent clear-cut abnormalities in global measures of serotonin signaling isn’t surprising if one considers the complexity of the receptors at which serotonin binds, the intricate neuroanatomical circuitry of the serotonin system, and the developmental role serotonin plays as a neurotrophic factor ^16– 18^. Many recent studies have focused on understanding the mechanisms through which serotonin affects depression by studying the impact of 5-HTT and the 15 known receptors through gene-association studies, human brain imaging, and pharmacological and genetic mouse models ^19^.

The success in treating depression by targeting the transporter with SSRIs prompted investigations into whether variability in 5-HTT expression levels could be involved in the etiology of depression. A highly cited study showed that there is an association between a polymorphism in the serotonin transporter (5-HTTLPR) and susceptibility to developing depression ^20^. This and other studies have shown that the short “s” allele, which results in lower levels of 5-HTT expression (at least *in vitro*) and therefore increased extracellular 5-HT, is associated with a *higher* risk of depression when combined with stressful life events ^21, 22^. This discovery would be unexpected if developmental considerations were not considered. Although inhibiting the function of the transporter during adulthood decreases depressive symptoms as in the case of SSRIs, reduced expression of 5-HTT during development may increase depressive behavior in adulthood. A human functional magnetic resonance imaging (fMRI) study supports this, showing that short allele carriers show morphological and functional alterations in limbic circuits ^23^. Additionally, mice lacking 5-HTT throughout life display increased depressive-like behaviors, and pharmacological blockade of 5-HTT in mice exclusively during early postnatal development resulted in increased adult depressive behavior ^24^. These results highlight the differences in developmental versus adult effects of altered serotonin neurotransmission on depression. 

In addition to the serotonin transporter, the majority of the 15 serotonin receptors have been implicated in the modulation of depression, depressive-like behaviors, or the response to anti-depressant treatment ^19^. There are numerous pre-clinical studies which have investigated the role of serotonin receptors using pharmacological manipulations and genetic knockout (KO) models in rodents ( Table 1). Given the breadth of this literature, this review will focus on two receptors that are among the most extensively studied for their role in modulating depression, the 5-HT _1A_ and 5-HT _1B_ receptor subtypes. In addition, attention will be paid to population-dependent and development-dependent effects of serotonin signaling at these receptors and will draw from both rodent and human studies.

**Table 1.** Preclinical evidence supporting the role for serotonin receptors in depression.

Receptor	PubMed Hits *	Pharmacological studies on depression	Genetic effects on depression	Other behavioral phenotypes
**5-HT _2A_**	588	Antagonists have antidepressant-like effects and potentiate the effects of SSRIs ^133, 134^	No known effect of 5-HT _2A_ KO on depressive-like behavior ^135^	Agonists are hallucinogenic; antagonists are antipsychotic and anxiolytic; KO mouse has reduced anxiety-like behavior ^135– 137^
**5-HT _2B_**	52	Agonists have antidepressant-like effects ^138^	Required for behavioral effects of SSRIs ^138, 139^	KO mouse shows increased impulsivity ^140^
**5-HT _2C_**	282	Antagonists have antidepressant-like effects; agonists have pro-depressive effects ^141, 142^	No known effect of 5-HT _2C_ KO on depressive-like behavior	Antagonists have anxiolytic effects; agonists decrease impulsivity and motivation for drug and food consumption; KO mouse has reduced anxiety-like behavior ^143– 145^
**5-HT _3A_**	252	Antagonist has antidepressant-like effects ^146^	5-HT _3_ required for exercise-induced antidepressant effects; KO has antidepressant-like phenotype ^147, 148^	Antagonists are anxiolytic ^149^
**5-HT _4_**	81	Agonists have rapid antidepressant-like effects ^150, 151^	KO has attenuated responses to stress ^152^	Agonists are anxiolytic; agonists improve cognitive performance and reduce feeding ^151, 153^
**5-HT _5A_**	5	Unknown	Unknown	KO mice display increased exploratory behavior ^154^
**5-HT _6_**	62	Agonists produce antidepressant-like effects and antagonists block the effects of SSRIs ^155, 156^	Unknown	Antagonists enhance cognitive performance; blockade of signaling is anxiogenic ^157, 158^
**5-HT _7_**	137	Antagonists have antidepressant-like effects ^159^	KOs have an antidepressant-like phenotype ^159^	Antagonists have pro-cognitive effects ^160^

*Number of PubMed hits based on the search terms including “depression” and the receptor as of August 25, 2016. N.B. 5-HT1D, 1E, 1F, 3B, and 5B are not included in the chart owing to a lack of published research concerning the role of these receptors in behavior. 5-HT, serotonin; KO, knockout; SSRI, selective serotonin reuptake inhibitor.

**Figure 1.**
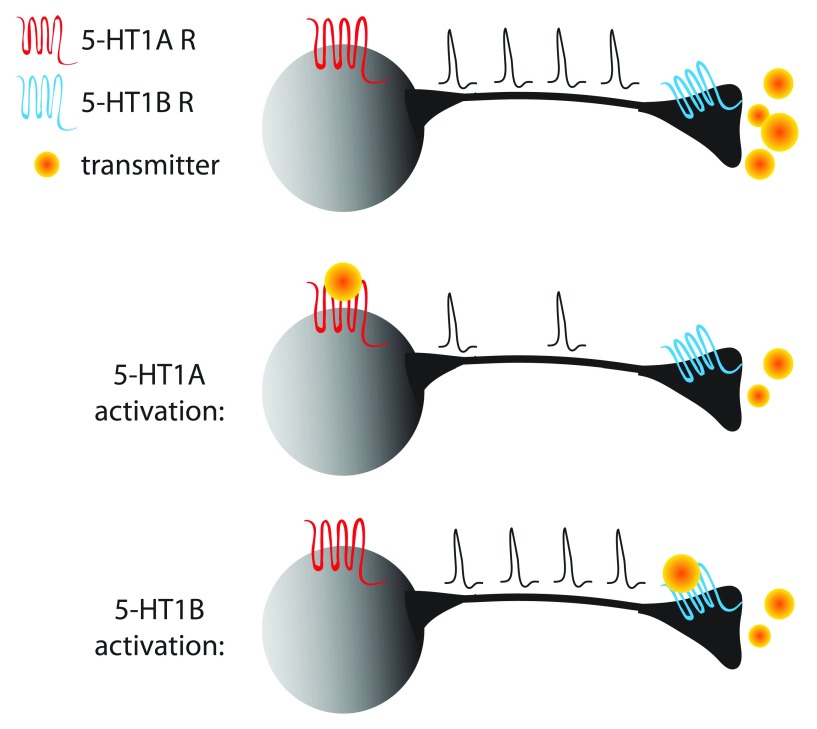
Schematic illustrating the inhibitory effects of serotonin (5-hydroxytryptamine, 5-HT) 1A (5-HT _1A_) (red) and 5-HT _1B_ (blue) receptors on the normal firing and neurotransmitter release of a neuron (top). Activation of 5-HT _1A_ receptors results in decreased firing (middle), while activation of 5-HT _1B_ receptors causes decreased neurotransmitter release through actions in the presynaptic terminal (bottom).

The 5-HT _1A_ and 5-HT _1B_ receptors are both inhibitory Gi/o-coupled seven transmembrane receptors that are located throughout the brain ^25– 27^. A major difference between these two receptors is their subcellular distribution ^28^. 5-HT _1A_ receptors are somatodendritic, while 5-HT _1B_ receptors are located on axon terminals ^27, 29^. This difference is also reflected in their mechanisms of inhibitory action ( Figure 1). Activation of either receptor causes decreased neurotransmitter release; however, 5-HT _1A_ receptor activation causes hyperpolarization, leading to decreased firing, while 5-HT _1B_ receptors inhibit voltage-gated calcium channels in the presynaptic terminal ^30– 32^. Another mechanism for 5-HT _1B_ receptor-mediated inhibition is via effects on 5-HTT, and activation of the 5-HT _1B_ receptor increases serotonin reuptake ^33, 34^.

**Figure 2.**
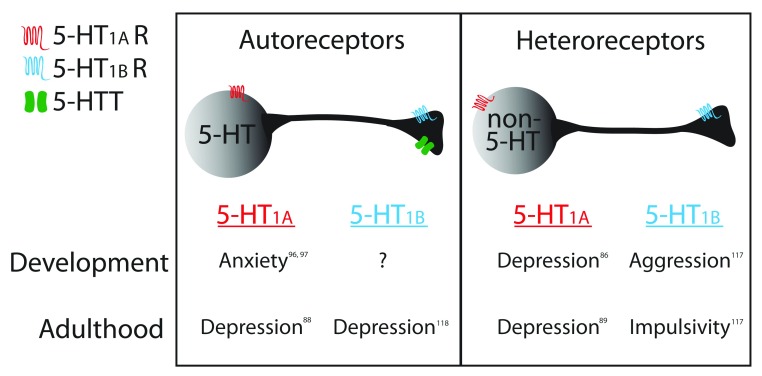
Diagram summarizing the roles of autoreceptor and heteroreceptor populations of serotonin (5-hydroxytryptamine, 5-HT) 1A (5-HT _1A_) and 5-HT _1B_ receptors on behavior during development and adulthood. 5-HTT, serotonin transporter.

Both 5-HT _1A_ and 5-HT _1B_ receptors act as autoreceptors located on serotonin neurons and also have heteroreceptor populations located on non-serotonin receptors ( Figure 2). Although the mRNA in the raphe (corresponding to autoreceptors) is comparable between the two receptors, their heteroreceptors have distinct patterns of expression ^35^. 5-HT _1A_ receptors are enriched in the hippocampus and cortex, while 5-HT _1B_ receptors are highly expressed in the basal ganglia ^36, 37^. These differences in mechanism of action and localization may play a role in the different functional effects of these receptors.

While this review focuses on the contribution of 5-HT _1A_ and 5-HT _1B_ receptors in depression and depressive-like behaviors, these receptors also modulate other psychiatric-relevant phenotypes. For example, alterations in 5-HT _1A_ receptor expression influence anxiety behavior, and 5-HT _1B_ receptor signaling affects reward- and impulsivity-related phenotypes. These receptor-based differences in serotonergic regulation of emotional behavior, which segment into endophenotypes, could contribute to the heterogeneity of symptoms found in MDD ^38^. Understanding the neural circuits that subserve these receptor-based and endophenotype-based differences can help clarify the often confusing and sometimes contradictory findings from various preclinical approaches. From a behavioral perspective, these phenotypes can be segmented through formal unsupervised factor analyses to better divide depressive behaviors into meaningful endophenotypes. Then predictors of the different endophenotypes could be tested by including genetic or pharmacological manipulations.

## 5-HT _1A_ and depression

Of the 15 known serotonin receptors, the 5-HT _1A_ receptor is the most studied for its role in depression ^39^. Quantification of 5-HT _1A_ receptor levels in humans from post mortem and positron emission tomography (PET) imaging studies reveals an increased level of 5-HT _1A_ receptors in patients diagnosed with MDD ^40– 42^. Gene association studies have linked a polymorphism in the 5-HT _1A_ regulatory region (rs6295; G-1019C) with receptor levels in the brain and also to increased risk for depression ^43– 47^. The GG genotype at this single nucleotide polymorphism (SNP) is associated with altered levels of 5-HT _1A_ receptor expression and reduced responsiveness to antidepressant treatment ^43, 48^. Additionally, clinical studies have revealed antidepressant effects of buspirone and other 5-HT _1A_ receptor agonists ^49, 50^.

Rodent models have also shown that 5-HT _1A_ receptor agonists, such as 8-hydroxy-2-(di-n-propylamino) tetralin (8-OH-DPAT), can have acute antidepressant-like effects ^51– 53^. These effects are blocked by 5-HT _1A_ receptor antagonists, suggesting that the antidepressant-like response is specific to 5-HT _1A_ receptor signaling ^54^. 5-HT _1A_ heteroreceptors, expressed throughout the limbic system, are the likely site of action for these acute 5-HT _1A_ receptor-mediated effects ^50, 55^. On the other hand, 5-HT _1A_ autoreceptors work in opposition to the heteroreceptors, leading to pro-depressive effects. Specifically, activation results in hyperpolarization and reduced firing of raphe neurons, leading to diminished serotonin release in projection regions ^56^. Therefore, stimulation of 5-HT _1A_ autoreceptors from increased extracellular serotonin following SSRI treatment is thought to oppose SSRI actions by downregulating serotonin neuron activity ^57^. Over the first few weeks of treatment, these receptors desensitize, which may underlie the delayed behavioral efficacy of SSRIs ^58^. Therefore, blocking 5-HT _1A_ autoreceptor activation has been introduced as an adjunctive therapy to SSRIs. 5-HT _1A_ receptor partial agonists such as pindolol, and more recently vilazodone, have been shown to be an effective adjunctive therapy to SSRIs in clinical studies ^59– 62^. The development of new agonists that preferentially target subpopulations of 5-HT _1A_ receptors, for example autoreceptors versus heteroreceptors, potentially through biased agonism, may be useful tools for the treatment of MDD ^63^.

Differences in receptor levels have also been modeled in mice by using genetic loss-of-function models and have allowed causal links between receptor expression levels and depressive-like behavior. 5-HT _1A_ receptor KO mice have an anti-depressive phenotype ^64, 65^. Tissue-specific KOs have been especially valuable for the dissection of this phenotype and have allowed investigations into the distinct roles of different populations of receptors ^66^. The absence of heteroreceptors results in increased depressive-like behavior- as measured in the forced swim test. This mouse model also allowed for temporal control of receptor expression, which revealed a developmental sensitive period for the effect of heteroreceptors on depressive-like behavior. Specifically, knockdown of 5-HT _1A_ heteroreceptors in adulthood was not sufficient to produce the depressive-like behavior. On the other hand, reduction of autoreceptors in adulthood increased mobility in the forced swim test, suggesting an “anti-depressed” phenotype.

Preclinical studies have also confirmed a causal role for alterations in 5-HT _1A_ receptor expression in antidepressant efficacy. 5-HT _1A_ receptor KO mice do not show a behavioral response to fluoxetine ^67^. As expected from the pharmacology work, this effect is not mediated by autoreceptors, since reduced expression of 5-HT _1A_ autoreceptors actually increases the speed and efficacy of SSRI response, requiring only 8 days of fluoxetine treatment to show a behavioral antidepressant-like response ^68^. Recent data show that 5-HT _1A_ heteroreceptors are critical for an effective behavioral response to an SSRI in mice ^69^. Genetic or viral deletion of 5-HT _1A_ receptors specifically in the dentate gyrus of the hippocampus reduced the behavioral response to fluoxetine. Furthermore, expression of 5-HT _1A_ receptors only in the dentate gyrus was sufficient for normal antidepressant-like responses. These results importantly demonstrate a mechanism for 5-HT _1A_-mediated antidepressant effects localized in the mature granule cells of the dentate gyrus.

## 5-HT _1A_ and other psychiatric-relevant phenotypes

Anxiety behavior is also modulated strongly by the 5-HT _1A_ receptor, and, among depressed patients, almost half have a comorbid anxiety disorder ^70^. In preclinical studies, 5-HT _1A_ receptor agonists have anxiolytic effects, and 5-HT _1A_ receptor KO mice display increased anxiety-like behavior ^64, 65, 71, 72^. The effect has a developmental sensitive period, since early developmental but not adult rescue of the receptor was sufficient to restore the normal phenotype in the KO ^73^. Consistent with this, early postnatal blockade of 5-HT _1A_ receptors, through genetic or pharmacological methods, also leads to increased anxiety ^74, 75^. Recent work has shown that the sensitive period is peri-pubertal, and tissue-specific KO mice point to a role for autoreceptors during this period of development ^66, 76, 77^.

Other psychiatric disorders have also been linked to the 5-HT _1A_ receptor, including bipolar disorder and post-traumatic stress disorder ^78, 79^. Additionally, the SNP rs6295 found in the premotor region that is associated with risk for depression is also linked with psychiatric hospitalization, a history of substance abuse, and prior suicide attempts ^43^. Consistent with the studies in depression, the G allele is associated with reduced expression of the 5-HT _1A_ receptor in the prefrontal cortex and an increased risk for psychiatric outcomes. Interestingly, the effects on receptor expression were also seen in the brain during early human embryonic development, suggesting its potential importance in mediating developmental contributions to adult depression. Finally, there were associations with childhood maltreatment with trends towards significant genotype by environment interactions ^40^.

## 5-HT _1B_ and depression

While the 5-HT _1B_ receptor is best known for its role in regulating aggressive and impulsive behavior, it also plays an important role in modulating depression. Activation of the 5-HT _1B_ receptor decreases serotonin levels in the brain through effects on release, synthesis, and reuptake ^33, 80, 81^. In humans, reduced 5-HT _1B_ receptor function is associated with MDD ^82^. Additionally, patients with MDD are less responsive to 5-HT _1B_ receptor agonists, suggesting reduced expression or desensitization ^83, 84^. This is consistent with clinical studies showing that 5-HT _1B_ receptor agonists produce antidepressant effects in humans ^85– 87^. This has also been shown in mice, with specific agonists resulting in antidepressant-like behavior ^88, 89^. However, genetic KO of the receptor also results in antidepressant-like behavior, suggesting that this is possibly caused by compensatory effects ^90– 94^.

Both autoreceptor and heteroreceptor populations of 5-HT _1B_ receptors have been implicated in depressive-like behaviors using rodent models. However, since 5-HT _1B_ receptors are located on presynaptic terminals, heteroreceptors and autoreceptors have overlapping localization ^95^. This rules out brain imaging and pharmacological manipulations in preclinical models as tools to differentiate the role of the two populations of receptors. Therefore, it has been only the recent availability of a tissue-specific genetic mouse model that has allowed the dissection of the role of 5-HT _1B_ receptors in the regulation of behavior ^96^.

Our recent studies show that selective ablation of 5-HT _1B_ autoreceptors results in decreased depressive-like behaviors in mice ^97^. These mice also show increased elevations in serotonin levels compared to controls following SSRI administration, suggesting a potential mechanism of action for the behavioral effects. Specifically, removing the terminal auto-inhibition may result in increased serotonin in projection regions that are relevant to depressive behavior. Furthermore, we also showed that the impact of 5-HT _1B_ autoreceptors on behavior was not due to developmental expression, since the phenotype was not recapitulated in a mouse with developmental knockdown. These data are consistent with other evidence suggesting a pro-depressive role for the activation of 5-HT _1B_ autoreceptors ^98, 99^. For example, 5-HT _1B_ mRNA is elevated in the raphe of rats following stress and in models of depression such as learned helplessness, and viral overexpression of 5-HT _1B_ receptors in the raphe results in depressive-like behavior following stress ^100^. In rats, reductions in 5-HT _1B_ receptor mRNA in the raphe are seen following SSRI treatment in post mortem brains ^101, 102^. This effect isn’t seen in other brain regions such as the cortex, hippocampus, or striatum, suggesting that this effect is specific to autoreceptors. Additionally, another study showed that 5-HT _1B_ autoreceptors may desensitize following SSRI treatment, similar to 5-HT _1A_ autoreceptors ^103^. Finally, a recent PET study in humans reported that following effective cognitive behavioral therapy for depression, 5-HT _1B_ receptor binding was reduced in the brainstem ^104^.

There is evidence which suggests an opposing role for 5-HT _1B_ heteroreceptors in depressive behaviors. Activation of 5-HT _1B_ heteroreceptors in a rodent serotonin depletion model (to remove the contribution of autoreceptors) results in an antidepressant-like effect ^105^. Additionally, reduced expression of 5-HT _1B_ heteroreceptors in the ventral striatum is associated with depression in humans ^82^. Finally, 5-HT _1B_ receptors located in the ventral striatum have been suggested to interact with p11 (a 5-HT _1B_ receptor-binding protein) to affect depression-related behaviors ^106, 107^.

## 5-HT _1B_ and other psychiatric-related phenotypes

Reward dysfunction is a major symptom of MDD which is mediated, in part, by altered signaling in the mesolimbic reward system ^108– 112^. 5-HT _1B_ receptors have been implicated in the neural basis of dysregulated reward sensitivity in a number of human studies and preclinical models ^113– 116^, and both 5-HT _1B_ receptor protein and mRNA are located within the mesolimbic pathway in the nucleus accumbens (NAc) and ventral tegmental area (VTA) ^95^. Additionally, activation of 5-HT _1B_ receptors in the VTA increases dopamine levels in the NAc, potentially via effects on GABAergic signaling in the VTA ^117^.

Many studies linking the receptor to functional deficits in reward processing have focused on addiction. Polymorphisms in the 5-HT _1B_ receptor gene have also been associated with drug and alcohol abuse ^118– 120^. Additionally, a PET imaging study revealed increased 5-HT _1B_ receptor binding in pathological gamblers, who have known deficits in reward sensitivity, and gambling disorder is highly comorbid with depression and alcohol and substance use disorders ^116, 121^. Another PET imaging study shows that there is reduced 5-HT _1B_ receptor binding in cocaine-dependent participants compared to in healthy controls ^122^. In preclinical models, 5-HT _1B_ receptor KO mice are more motivated to self-administer cocaine ^123^. Consistent with this, 5-HT _1B_ receptor agonists attenuate the motivation for cocaine but paradoxically increase the rewarding effects of cocaine ^124^. These effects are mediated by 5-HT _1B_ receptor expression on medium spiny neurons in the NAc, likely through their projections to the VTA ^125, 126^. Additionally, 5-HT _1B_ receptors are required for the rewarding properties of social interaction, supporting an impact on general reward systems ^114^.

5-HT _1B_ receptors are also implicated in impulsive aggression. In humans, polymorphisms in the gene encoding 5-HT _1B_ receptors have been associated with aggression, suicide, and disorders that include impulsivity as a core phenotype, including attention deficit hyperactivity disorder and substance use disorder ^115, 120, 127^. In mice, 5-HT _1B_ receptor KOs are highly aggressive in tests of male and female aggression and also display increased impulsivity ^128– 130^. Additionally, 5-HT _1B_ receptor agonists are known as “serenics” because they decrease aggression ^131^. While the aggressive and impulsive phenotype was originally thought to be modulated by the same underlying circuits, our recent work shows that distinct populations of 5-HT _1B_ receptors modulate aggression and impulsivity ^96^. Furthermore, developmental expression of the 5-HT _1B_ receptor influences aggression, while adult expression modulates impulsive behavior.

## Conclusion

There is a considerable body of research that implicates serotonin in the modulation of depression and depression-related behaviors. The preclinical work delineating the effects of signaling through the 5-HT _1A_ and 5-HT _1B_ receptors has been made possible because of careful pharmacological studies as well as the development of transgenic mouse models that have allowed for tissue-specific and inducible knockdown. These studies have highlighted the complexity of serotonin receptors, showing that their role varies through the lifespan and by cell-type population. Additionally, the availability of specific radioligands for PET imaging of these receptors has allowed for the translation of findings from preclinical work to humans. The large number of studies concerning the role of these receptors is partially due to the fact that the 5-HT _1_ receptor subtypes were some of the first discovered, and it may be only a matter of time before the roles of more newly discovered receptors are clarified ^17^.

Despite the amassing of evidence of serotonin receptor-specific involvement in depression, the primary pharmaceutical treatment strategy for depression remains the inhibition of serotonin reuptake. The lack of new treatment options is surprising given the need for them, since current SSRI treatments are ineffective in one-third of patients ^132^. Additionally, the majority of patients, as seen in the STAR*D study, don’t respond to administration of the first SSRI treatment, requiring multi-step treatment plans that take months ^132^. Furthermore, the considerable differences in treatment outcome also emphasize the heterogeneity of the depressed patient population. A better understanding of receptor signaling and neural circuit mechanisms by which serotonin affects depression may inform the development of novel, more targeted drugs that influence specific receptors, signaling cascades, or time periods. Also, personalized treatment plans could be developed based on symptoms, biomarkers, or pathophysiological presentation.
